# Higher Neck Pain Intensity and the Presence of Psychosocial Factors Are More Likely When Headache is Present after a Whiplash Injury: A Case-Control Study

**DOI:** 10.1093/pm/pnac038

**Published:** 2022-02-25

**Authors:** Ernesto Anarte-Lazo, Carlos Bernal-Utrera, Juan Montaño-Ocaña, Deborah Falla, Cleofas Rodriguez-Blanco

**Affiliations:** Doctoral Program in Health Sciences, University of Seville, Seville, Spain; Centre of Precision Rehabilitation for Spinal Pain (CPR Spine), School of Sport, Exercise and Rehabilitation Sciences, University of Birmingham, Birmingham, UK; Physiotherapy Department, Faculty of Nursing, Physiotherapy and Podiatry, University of Seville, Seville, Spain; Musculoskeletal Pain and Motor Control Research Group, Faculty of Health Sciences, Universidad Europea de Madrid, Villaviciosa de Odon, Madrid, Spain; Department of Physiotherapy, Faculty of Biomedical and Health Sciences, Universidad Europea de Madrid, Villaviciosa de Odon, Madrid, Spain; Centre of Precision Rehabilitation for Spinal Pain (CPR Spine), School of Sport, Exercise and Rehabilitation Sciences, University of Birmingham, Birmingham, UK; Physiotherapy Department, Faculty of Nursing, Physiotherapy and Podiatry, University of Seville, Seville, Spain

**Keywords:** Whiplash Associated Disorders, Headache, Psychosocial Factors, Neck Pain, Rehabilitation, Disability

## Abstract

**Background:**

Several factors such as neck pain intensity, disability, anxiety, depression, female sex, or a previous history of headache are associated with post-whiplash headache. However, the possible role of psychosocial factors contributing to the presence of headache or worsening of headache after a whiplash trauma remains unclear. To address this gap in knowledge, there is the need to assess psychosocial factors concerning headache shortly after a whiplash injury.

**Objective:**

To evaluate psychological features, pain and disability in people with acute whiplash associated disorders (WAD) and compare these features between those with and without headache.

**Design:**

Case-control study.

**Setting:**

A secondary care traumatology center.

**Methods:**

Forty-seven people with acute WAD were recruited; 28 with headache, and 19 without. All participants completed self-reported questionnaires including Visual Analogue Scale (VAS) for neck pain intensity, the Neck Disability Index (NDI), Pain Catastrophizing Scale (PCS), Tampa Scale Kinesiophobia-11 (TSK-11), and State-Trait Anxiety Inventory.

**Results:**

Neck pain intensity (*P *< .001), neck disability (*P *< 0.001), pain catastrophizing (*P *< .001), kinesiophobia (*P *< .001), and anxiety state (*P *= .007) and trait (*P *= .05) were higher in those with headache when compared to those without. In addition, high levels of neck pain (*P *= .025), moderate levels of neck disability (*P *< .001), moderate levels of pain catastrophizing (*P *= .015), and moderate (*P *= .002) and severe (*P *= .016) levels of kinesiophobia were related to the presence of headache.

**Conclusions:**

The level of neck pain intensity and disability, kinesiophobia, catastrophizing, and anxiety were all greater in people with acute WAD who presented with a headache compared to those without headache.

## Introduction

A whiplash injury is defined as the acceleration-deceleration mechanism that produces a transfer of energy to the neck, usually provoked by a rear-end car collision, which can lead to a variety of symptoms and clinical manifestations, known as whiplash associated disorders (WAD) [1, 2]. Whiplash injuries are common and contribute substantially to the impairment and disability that results from traffic injuries [3]. It has been estimated a prevalence of up to 400 cases per 100,000 inhabitants per year in Europe [4].

According to the International Headache Society (IHS), “headache attributed to whiplash” is a headache that appears within 7 days after the accident, or a previous headache that worsens in this period of time after the accident [5]. Indeed, headache is one of the most common symptoms after a whiplash injury [6]. The prevalence of headache is reported to be up to 60%, 7 days after the accident, and 23%, 30%, and 38% at 3, 6, and 12 months, respectively, and is one of the most common symptoms after neck pain [4, 6]

Several factors such as neck pain intensity, disability, anxiety, depression, female sex, or a previous history of headache are associated with post-whiplash headache [7–9]. However, the possible role of psychosocial factors contributing to the presence of headache or worsening of headache after a whiplash trauma remains unclear. To address this gap in knowledge, there is the need to assess psychosocial factors concerning headache shortly after a whiplash injury. The aim of the present study was to analyze neck pain and headache intensity, neck disability, kinesiophobia, pain catastrophizing, and anxiety in the short term after a whiplash trauma, comparing differences between those who develop headache or experience worsened headache compared to those who do not. We hypothesized that those with headache would demonstrate greater psychosocial features, pain, and disability.

## Methods

### Study Design

A case-control study was carried out involving patients with acute pain attributed to a whiplash injury due to a traffic accident who were attending the Clinica San Vicente, Madrid, Spain, from September 2020 to February 2021. Ethical approval was granted by the applicable institutional human research ethics committee from University Rey Juan Carlos, Madrid, Spain (Ref: 1003202108121). All participants gave their written informed consent to participate in this study. The study was conducted according to the Declaration of Helsinki and is reported in accordance with STROBE guidelines [10, 11]

### Participants

Consecutive patients with a diagnosis of WAD were recruited from the Traumatology Department of the Clinic. By the nature of the Traumatology Clinic, we only collected victims of the accidents. After being diagnosed by a physician, who informed patients about the study, those who agreed to participate provided written informed consent and were referred to the Physiotherapy Department.

Inclusion criteria consisted of Grade II WAD, as defined by The Quebec Task Force on Whiplash-Associated Disorders [1, 2] between 7 and 30 days after the accident, and aged between 18 and 65 years old. Individuals were excluded if they suffered from previous headache that did not increase after the accident (as considered by the International Headache Society) [5] were diagnosed with fibromyalgia or had a history of generalized pain, had experienced a previous whiplash injury, had a diagnosed temporomandibular disorder (TMD), had been diagnosed with osteoporosis, cervical myelopathy, vertebral fractures and/or, inflammatory or rheumatic diseases, had a known psychological disorder or congenital disturbances, had undergone previous surgery in the cervical region, had received physical therapy treatment after the accident but before participation in the study, or were not able to complete patient-reported outcome measures. In addition, with the aim of excluding those subjects suffering from concussion, we followed the criteria of the International Headache Society, and we excluded subjects if they had experienced one or more of the following signs and/or symptoms: confusion, disorientation or impaired consciousness; loss of memory for events immediately before or after the accident; and one or more of the following: nausea, vomiting, visual disturbances, dizziness and/or vertigo, gait and/or postural imbalance, and impaired memory and/or concentration. The participants were categorized into two groups: “Cases” were those with acute WAD with concomitant headache related to the traumatic event, while “controls” were those with acute WAD but without headache.

The sample size estimation was performed using the Grammo calculator v.7.12. Based on the analysis of variance of means and estimating an alpha risk of 5% (0.05), a beta risk of 20% (0.20), a unilateral contrast, a typical deviation of 10% (0.10), a minimum clinical difference of 20% (0.2) in Neck Disability Index in neck pain disorders [12] and assuming no dropouts due to the design of the study, at least 18 participants are required (nine per group).

### Procedures

Upon recruitment by the physician, participants were advised not to disclose their headache status when attending the Physiotherapy Department for assessment and therefore the assessor was blinded to group allocation. Before the evaluation, participants were advised that these questionnaires were not going to be considered for their final health report generated by both Physiotherapy and physician departments and therefore would not be reviewed as part of any insurance claim. All participants completed questionnaires related to psychosocial features and neck pain and disability as detailed below.

### Outcome Measures

Age, sex, height, weight, type of previous headache (if present), and occupation were recorded for all participants.

*Visual Analogue Scale (VAS).* Neck pain and headache intensity was assessed using the VAS, with a score varying from 0 to 100 (0 = no pain; 100 = worst pain imaginable) [13], which has established good reliability [14].

*Neck Disability Index (NDI).* The Spanish version of the NDI was used to evaluate neck disability (internal consistency Chronbach’s alpha 0.80; excellent reliability ICC (95% confidence interval [CI]) = 0.88 [0.63 to 0.95]; good construct validity when compared to Global Rating of Change (*P* < .001)) and is commonly used to assess disability in people with WAD [15–17].

*Tampa Scale Kinesiophobia-11 (TSK-11).* The Spanish version of the TSK-11 was used to measure fear of movement (internal consistency Chronbach’s alpha 0.79). This tool is composed of 11 items and scores range from 1 (strongly disagree) to 4 (completely agree). The total score can range from 11 to 44, with higher scores reflecting greater kinesiophobia. The TSK-11 has good test-retest reliability (ICC (95% CI) 0.81 (0.71 to 0.88)) and a highly significant correlation with change scores on the TSK (r = 0.93, *P* < .001) [18, 19].

*Pain Catastrophizing Scale (PCS).* The Spanish version of the PCS was used (internal consistency Cronbach’s alfa 0.79, good test-retest reliability ICC 95% CI = 0.84) [20]. This scale is composed of 13 items and the total score ranges from 0 to 52, with higher scores reflecting greater levels of pain catastrophizing. Good convergent validity was observed with a high correlation with the Fear Avoidance Belief Questionnaire (r = 0.66).[20]

*The State-Trait Anxiety Inventory.* The Spanish version of this questionnaire (internal consistency Cronbach’s alfa 0.92; test-retest reliability with an ICC of 0.80 (95% CI: 0.66 to 0.89) was used to assess anxiety; good construct validity since negative correlation was found with Short Form Health Survey-12 mental health (rho=−0.6752) [21–23]. This questionnaire comprises two different scales of 20 items each and scores range from 0 to 60, with higher scores indicating higher anxiety.

### Data Analysis

The statistical analysis was carried out using IBM-SPSS Statistics 24 software. The Kolmogorov-Smirnov test was applied to test the distribution of the data. Student’s *t*-test was applied for paired variables when the data had a parametric distribution. The Mann-Whitney *U* test was used to analyze data with a non-parametric distribution. The χ^2^ test was used to compared qualitative variables. Independent variables were age, sex, height, weight, and days from the accident to evaluation. The correlation of variables was performed through odds ratio; dependent variables were classified as mild, moderate and severe: VAS (0–40, 41–70, 71–100), NDI (0–20, 21–40, 41–50), TSK-11 (11–22, 23–33, 34–44), PCS (0–17, 18–35, 36–54), Anxiety State and Trait (0–20, 21–40, 41–60). The confidence level used was 95% (0.05), and the power of the study was 90% (0.1).

## Results

**Figure 1.**
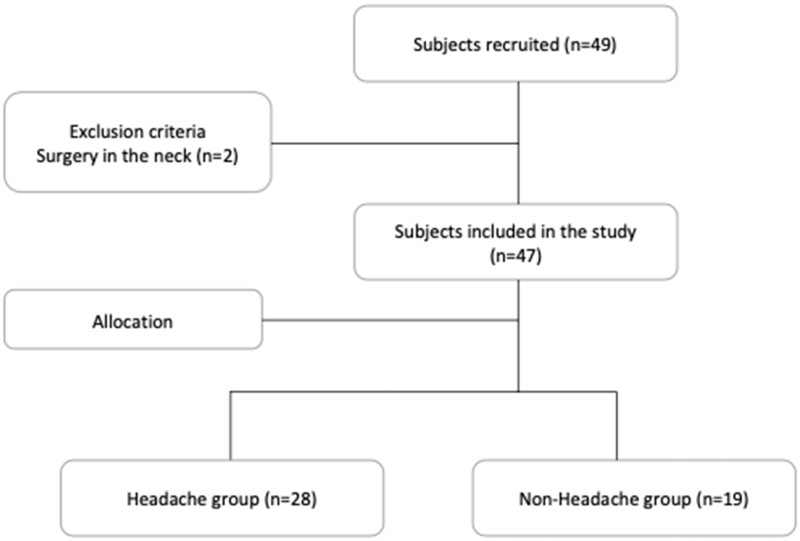
Flowchart of the selection procedure.

Forty-nine people were recruited and, after the exclusion of two, 47 patients remained and participated in the study (Figure 1). Among them, 28 participants (59.6%; 16 female) presented with headache and had a mean age of 37.6 years (standard deviation [SD]: 11.1 years). Five of these patients suffered previous headaches which had increased after the whiplash injury: two presented with migraine (one episodic and one chronic) and three tension-type headache (one episodic and two chronic). Nineteen (40.2%; 5 female) were considered as controls due to the absence of headache, and they had a mean age of 40.9 years (SD: 10.9 years). In the headache group, the mean (SD) in height (cm) and weight (kg) were 174.5 (8.8) and 70.7 (10.1), respectively, and the median (SD) days from the accident until the assessment was 13.4 (4.3); in the no-headache group, mean height and weight were 177.1 (9.9) and 76.6 (10.4), respectively, and the median days from the accident until the assessment was 11.7 (3.7). No significant differences between groups were found for age, height, weight or days from the accident to the assessment. A larger proportion of men were in the no-headache group (Table 1). Regarding their occupation, nine participants had administrative roles, three were lawyers, four teachers, six students, four health-workers, three engineers, five executives, one soldier, one basketball player, one policeman, one bricklayer, one taxi driver, one architect, one plumber, one librarian, and five were unemployed (Rev2.P4).

**Table 1.** Sociodemographic features of the participants with and without headache

Variables	Group		Z
	Headache (n = 28)	No Headache (n = 19)	*P*
Age (i)	37.6 ± 11.1	40.9 ± 10.9	.319*
Gender (ii)	12/16	14/5	—
(male, female)			
Height (i)	174.5 ± 8.8	177.1 ± 9.9	.370*
(cm)			
Weight (i)	70,7 ± 10,1	76.6 ± 10.4	.064*
(Kg)			
Days (i)	13,4 ± 4,3	11.7 ± 3.7	.152*

* T Student; (i) Data expressed as means ± standard deviation; (ii) Data expressed as percent (partial/total).

Z = Shapiro-Wilk Normality Test; *P* = statistical significance.

For the dependent variables, significant differences were found between groups for neck pain intensity (VAS), NDI, TSK-11, PCS, and Anxiety State (all *P* < .01) and Trait (*P* = .05) (Table 2).

**Table 2.** Neck pain intensity and disability and psychosocial variables presented for those with and without headache

Variables	Group		Z
	Headache (n = 28)	No Headache (n = 19)	*P*
VAS neck (i)	61.2 ± 14.5	35.5 ± 14.4	.001*^,†^
(mm)			
VAS headache(i)	47.4 ± 14.2	—	—
(mm)			
NDI (i)	24.0 ± 7.5	12.4 ± 4.4	.001*^,†^
TSK-11 (i)	29.6 ± 7.4	19.1 ± 4.8	.001*^,†^
PCS (i)	20.7 ± 11.0	5.6 ± 4.0	.001*^,‡^
Anxiety state (i)	26.0 ± 5.9	21.7 ± 4.4	.007*^,‡^
Anxiety trait (i)	25.9 ± 5.4	23.0 ± 4.3	.05*^,†^

* T Student; ^†^U Mann-Whitney; ^‡^Chi-Square; (i) Data expressed as means ± standard deviation; (ii) Data expressed as percent (partial/total).

VAS = Visual Analogue Scale; NDI = Neck Disability Index, TSK-11; Tampa-Scale Kinesiophobia 11; PCS = Pain Catastrophizing Scale; Shapiro-Wilk Normality Test; *P* = Statistical Significance

* Indicate statistically significant differences between groups (*P* < 0.05).

Table 3 presents the frequency distribution of the presence of headache with regards to the severity of each of the other variables (VAS, NDI, TSK-11, PCS, and Anxiety). Our results revealed a relationship between the presence of headache and (a) the presence of severe neck pain (>70 mm VAS), being 8 times more probable; (b) presenting with moderate neck disability (NDI = 21–40), being 34.5 times more probable; (c) the presence of moderate levels of catastrophizing (PCS = 18–36), being 10 times more probable, (d) the presence of moderate and severe levels of kinesiophobia (TSK-11 = 22–33/34–44), being 15 and 10 times more probable, respectively. We also found an inverse correlation for the same variables, that is, low levels of neck pain, neck disability and pain catastrophizing implied that people were 4 times less likely to suffer from headache. A trend only was observed for the measures of State and Trait Anxiety.

**Table 3.** Association analysis of the level of risk for the presence of headache

Variable	χ^2^	*P*	OR	95% CI	95% CI
				Lower Limit	Upper Limit
Neck Pain—Mild	12.05	.001*	0.087	0.19	0.39
Neck Pain, Moderate	1.57	.21	2.13	0.65	6.95
Neck Pain, Severe	4.99	.025*	8.57	0.99	73.94
NDI, Mild	19.54	.001*	0.25	0.003	0.21
NDI, Moderate	17.78	.001*	34.55	4.06	293.98
NDI, Severe	0.81	.78	1.43	0.12	16.86
TSK, Mild	23.32	.001*	0.03	0.004	0.148
TSK, Moderate	9.17	.002*	15.6	1.82	133.4
TSK, Severe	5.86	.016*	10.0	1.16	86.45
PCS, Mild	10.45	.001*	0.06	0.01	0.48
PCS, Moderate	5.96	.015*	10.00	1.17	85.59
PCS, Severe	1.69	.19	4.00	0.43	37.05
AnxietyS, Mild	3.18	.074	0.27	0.06	1.21
AnxietyS, Moderate	3.18	.074	3.85	0.83	17.93
AnxietyS, Severe	Constant				
AnxietyT, Mild	0.48	.49	0.61	0.15	2.48
AnxietyT, Moderate	0.48	.49	1.64	0.40	6.70
AnxietyT, Severe	Constant				

χ**^*2*^** = Pearson Chi**^*2*^** Test; OR = Odds Ratio; CI = Confidence Interval; *P* = *P* values; statistical significance; NDI = Neck Disability Index; TSK = Tampa Scale Kinesiophobia; PCS = Pain Catastrophizng Scale; AnxietyS = Anxiety State; AnxietyT = Anxiety Trait.

We also performed both type of analyses by excluding those patients from headache group who presented a previous headache condition (n = 5). This, however, did not influence the results.

## Discussion

This is the first study to assess differences in psychological features, neck pain intensity and disability between patients with acute WAD grade II presenting either with or without headache. Our findings demonstrate that the level of neck pain and disability, kinesiophobia, and catastrophizing are greater in those patients with WAD who have headache.

It has been reported that headache can occur in up to 60% of people shortly after a whiplash injury [6]. Our findings concur with this report since 59.6% of our participants presented with headache in the acute phase (average 13.4 days after the whiplash injury).

The influence of psychosocial factors on symptoms in people with WAD has been frequently evaluated [24, 25] and studies have shown that features such as kinesiophobia and pain catastrophizing are associated with the course of neck symptoms following a whiplash injury [26]. The degree of pain catastrophizing and kinesiophobia in addition to other psychological factors, have shown to have prognostic value of poor recovery from whiplash injury [27]. Although not studied specifically in people with a whiplash injury, the extent of psychosocial factors affects the transition from acute to persistent post-traumatic headache [28]. Post-whiplash symptoms, including headache, have been partially attributed to the presence of central sensitization and the presence psychological factors [29].

Unique to the current study, we found that the presence of high levels of neck pain, moderate levels of neck disability and pain catastrophizing, and moderate/severe levels of kinesiophobia were associated with the presence of headache. Psychological features can be evident in many patients with WAD and, according to our results, especially in those with headache. Since treatments addressing these factors have shown promising results [30], and psychological factors such as pain catastrophizing mediates the outcome of physical therapy treatments [31], translating these findings to clinical practice could help clinicians to adopt management strategies according to the needs of individual patients [32]. Clinicians should consider the likelihood of greater psychological features in people with acute WAD grade II who present with headache. These findings may facilitate clinical reasoning and facilitate the challenge of providing the right intervention, for the right person, at the right time [33].

### Methodological Considerations

It is relevant to note that the participants completed the questionnaires at the Traumatology Clinic which is where they were going to be evaluated for possible economic compensation; although they were advised that the information collected in our study was not related to their legal case and would not be used, this still may have influenced their answers. Additionally, this study only evaluated pain intensity, disability, psychological factors, and headache status in the short term. Future studies should address the interaction between all these factors and long-term health status.

A further consideration is the sample size and a larger sample size may have resulted in the trend observed for anxiety to become a significant result. Previous work has shown that anxiety is among several psychological variables which strongly predict the transition from acute to chronic pain [34, 35] as well as the outcome following physical therapy treatments [36].

Moreover, although our exclusion criteria were developed to avoid the inclusion of patients with concussion, this condition is highly variable in its presentation and usually very similar to whiplash symptomatology. Therefore, some patients with mild concussion could be included in our sample [37].

Finally, we did not collect data regarding TMD symptomatology, which can be common in people who have sustained a whiplash injuries and for people with headache [38, 39].

## Conclusion

The level of neck pain intensity and disability, kinesiophobia, catastrophizing, and anxiety were all greater in people with acute WAD grade II who presented with a headache compared to those without headache. People with headache were eight times more likely to have severe neck pain, more than 34 times more likely to have at least moderate neck disability, 10 times more likely to have moderate levels of catastrophizing and more than 10 times more likely to have moderate or severe levels of kinesiophobia. Further research is encouraged to determine whether the presence and extent of headache influences longer term outcome.

## Ethical Approval

Ethical approval was granted by the applicable institutional human research ethics committee from University Rey Juan Carlos, Madrid, Spain (Ref: 1003202108121).

## Authors’ Contributions

E.A.L. was the lead researcher, formulated the focus of the study and conducted the study. C.B.U. performed the statistical analysis. J.M.O. contributed to manuscript development. D.F. contributed to the formulation of the study and the development of the study. C.R.B. was the director of the study and contributed to statistical analysis. E.A.L. drafted the manuscript, and C.B.U., J.O.M., D.F., and C.R.B. revised the manuscript. All authors approved the final version of the manuscript.

## Supplementary Material

pnac038_Supplementary_Data

## Data Availability

The data are available upon request to the corresponding author.

## Notes

Funding sources: Authors declare no funding source.
